# Effects of three blood purification methods on serum fibroblast growth factor-23 clearance in patients with hyperphosphatemia undergoing maintenance hemodialysis

**DOI:** 10.3892/etm.2014.1543

**Published:** 2014-02-13

**Authors:** LI-YING MIAO, BIN ZHU, XIAO-ZHOU HE, JIN-FENG LIU, LI-NA JIN, XIU-RONG LI, LI-NA XUE, TIAN HUANG, JIAN-QIN SHEN, CHANG-YING XING

**Affiliations:** 1 The Blood Purification Center, The Third Affiliated Hospital of Soochow University, Changzhou, Jiangsu 213003, P.R. China; 2 Department of Critical Care Medicine, The Third Affiliated Hospital of Soochow University, Changzhou, Jiangsu 213003, P.R. China; 3 Department of Urology, The Third Affiliated Hospital of Soochow University, Changzhou, Jiangsu 213003, P.R. China; 4 Department of Nephrology, The First Affiliated Hospital of Nanjing Medical University, Nanjing, Jiangsu 210029, P.R. China

**Keywords:** hyperphosphatemia, fibroblast growth factor-23, hemodialysis, hemodiafiltration, hemoperfusion

## Abstract

The aim of the present study was to investigate the effects of three blood purification methods on fibroblast growth factor-23 (FGF-23) clearance in patients with hyperphosphatemia undergoing maintenance hemodialysis (MHD). In addition, the correlation between serum FGF-23 and phosphorus (Pi) levels and the clinical implications were identified. Sixty-five MHD patients with hyperphosphatemia were randomly divided into three groups: Hemodialysis, HD (n=23); hemodiafiltration, HDF (n=21); and hemodialysis+hemoperfusion, HD+HP (n=21) groups. Serum Pi, FGF-23, blood urea nitrogen, serum creatinine and associated bio-marker levels were measured prior to and following treatment. The expression level of serum FGF-23 was observed to be positively correlated with Pi (r=0.45, P<0.01). The three blood purification methods that were adopted for the present study exhibited significant and effective clearance of serum Pi (P<0.05). The post-treatment serum FGF-23 levels were significantly decreased in the HDF and HD+HP groups (P<0.05). Therefore, HDF may be an effective method for clearing serum FGF-23 in MHD patients exhibiting hyperphosphatemia.

## Introduction

Chronic kidney disease (CKD) is characterized by the presence of kidney damage, such as albuminuria and/or decreased kidney function, for example, a glomerular filtration rate (GFR) <60 ml/min/1.73 m^2^ for ≥3 months (1–3). Furthermore, CKD is a life-threatening disorder, affecting a small number of people who subsequently require care from a nephrologist. In the USA, the prevalence to incidence ratio of CKD is currently ~200:1 (4). When symptoms of CKD are severe, it is termed end-stage renal disease (ESRD), the treatment of which, is via renal replacement therapy (RRT) or transplantation. The complications of ESRD include increased risk of cardiovascular disease, infection, cognitive impairment and impaired physical function; however, CKD and metabolic bone disorders (CKD-MBD) are the predominant complications (5–8). The clinical manifestations of CKD-MBD include imbalances in blood calcium (Ca) and phosphorus (Pi), vitamin D deficiency, increased levels of parathyroid hormone (PTH) and serum fibroblast growth factor-23 (FGF-23) (9). Moreover, CKD-MBD results in bone damage, vascular calcification and an increased risk of cardiovascular events, which consequently, may be associated with dialysis quality, quality of life and mortality (10).

A previous study by Slatopolsky and Moe (11) demonstrated that hyperphosphatemia was critical in the progression of CKD-MBD, as well as identifying that hyperphosphatemia was closely associated with the initiation, progression and deterioration of CKD-MBD. A recent study by Shigematsu *et al* (12) demonstrated that various methods of treatment were required in the clinical management of hyperphosphatemia, as dietary Pi restriction and Pi removal via hemodialysis alone were insufficient.

FGF-23, an important regulatory cytokine, is a hormone found in the blood that controls phosphate metabolism, which ultimately influences the prognosis of patients exhibiting CKD. FGF-23 is predominantly produced and secreted by osteogenic cells and osteoblasts, with the kidney as the primary target organ. During the early stages of CKD, FGF-23 may contribute to maintaining the serum Pi levels within the normal range by increasing the renal excretion of Pi (13). However, Bia *et al* (14) demonstrated that when kidney function declined, the serum levels of FGF-23 increased, whereas the Pi values were not considered to be abnormal until the estimated (e)GFR in the maintenance hemodialysis (MHD) patients decreased to <30 ml/min/1.73 m^2^. In addition, a study has demonstrated that there may be a negative feedback loop, which exists between FGF-23 levels and Pi disorders (9).

However, effective removal of FGF-23 in MHD patients exhibiting hyperphosphatemia by blood purification is a complex issue and currently there are only a small number of studies regarding it. In the present study, three different blood purification methods; hemodialysis (HD), hemodiafiltration (HDF), and hemodialysis and hemoperfusion (HD+HP), were adopted to compare the clearance efficacy of FGF-23 in MHD patients exhibiting hyperphosphatemia.

## Patients and methods

### Patients

Sixty-five MHD patients (37 males and 28 females) with hyperphosphatemia who had received RRT in the Blood Purification Center of The Third Affiliated Hospital of Soochow University (Changzhou, China) were enrolled in the present prospective, randomized controlled study. Written informed consent was obtained from all participants and the present study was approved by the Ethics Commission of Soochow University (Changzhou, China).

Patients with severe heart, liver and infectious diseases were excluded from the study and the participants with the following primary diseases were enrolled: Chronic glomerulonephritis (n=35), hypertensive nephropathy (n=7), diabetic nephropathy (n=9), obstructive nephropathy (n=2), polycystic kidney disease (n=5), systemic lupus erythematosus (n=2) and others (n=5). The diagnostic criteria of hyperphosphatemia were established according to the National Kidney Disease Foundation-Kidney Disease Outcomes Quality Initiative (NKF-K/DOQI); a pre-dialysis serum phosphorus level ≥1.78 mmol/l indicated hyperphosphatemia (15).

The 65 participants were randomly divided into three groups: The HD group, n=23 patients; the HDF group, n=21 patients and the HD+HP group, n=21 patients.

### Blood purification methods

The patients in the HD group were treated using low flux synthetic filters (Diacap LOPS 15; B. Braun, Melsungen, Germany) and high flux synthetic filters (Diacap HIPS 15; B. Braun) were utilized on the patients in the HDF group. The duration of the HD and HDF treatments for the two groups was 4 h and a blood flow rate of 200–250 ml/min was used. HDF was conducted using a post-dilution replacement fluid with a volume of 30% of the ultrafiltration blood flow. The procedure in the HD+HP group was conducted over 4 h, which was divided into two parts; initially, the HA130-type resin HP (Jafron Biomedical Co., Ltd, Zhuhai, China) was connected in series prior to the Diacap LOPS 15 for the first 2 h. This was followed by the exclusive use of Diacap LOPS 15 for the subsequent 2 h; the blood flow rate was 180–250 ml/min. Bicarbonate dialysate was administered to the three groups with a 500 ml/min flow rate and heparin served as the anticoagulant.

### Blood samples and biochemical analysis

Blood samples were collected from the arterial blood line immediately prior to and following the RRT sessions. The levels of blood urea nitrogen (BUN), serum creatinine (SCr), serum Ca and Pi of the patients were assessed using an automatic biochemical analyzer (Hitachi, Ltd., Tokyo, Japan). FGF-23 was analyzed via enzyme-linked immunosorbent assay (ELISA) and the reagents were provided by Shanghai BlueGene Biotech Co., Ltd. (Shanghai, China). To measure the serum FGF-23 concentration, the blood samples were immediately centrifuged (1,000 × g for 15 min) and the serum was stored at −80°C until all of the samples had been collected. The FGF-23 was analyzed in accordance with the following instructions.

### Analysis of FGF-23 levels

All of the reagents and samples were heated to room temperature prior to use. The standards or samples (100 μl of each) were added to the appropriate wells in the polyclonal and sheep anti-FGF-23 antibody pre-coated microtiter plate (Shanghai BlueGene Biotech Co., Ltd., Shanghai, China). Conjugate (50 μl) was added to each well and mixed and the plate was covered and incubated for 1 h at 37°C. The microtiter plate was washed five times using a washing machine and blotted dry using absorbent paper. Substrate A (50 μl) and 50 μl substrate B were subsequently added to each well, the plate was covered and incubated for 10–15 min at 20–25°C (exposure to sunlight was avoided). A stop solution (50 μl) was added to each well and mixed and the optical density was determined at a wavelength of 450 nm using a microplate reader.

### Calculations

Serum Pi and FGF-23 reduction ratios were calculated using: i) The urea reduction ratio (URR) equation: URR=(C_pre_-C_post_)/C_pre_; where, C_pre_ and C_post_ are pre- and post-treatment BUN concentrations, respectively, and ii) the urea clearance index (Kt/V): Kt/V=−ln(R-0.008xt)+(4-3.5xR)xUF/W where; ln, natural logarithm; R, post-/pre-BUN ratio; t, dialysis session length (hours); UF, ultrafiltrate volume (liters); W, post-dialysis weight (kg) (16).

### Statistical analysis

The data were expressed as means ± standard deviation. Statistical analysis was performed with SPSS 13.0 (SPSS Inc., Chicago, IL, USA). The statistical significance of pre- and post-treatment differences was analyzed using paired t-tests. One-way analysis of variance was applied to compare the three groups and correlation analysis between FGF-23 and Pi levels was conducted using Pearson’s product-moment coefficient. P<0.05 was considered to indicate a statistically significant difference.

## Results

### Patient characteristics

A total of 65 patients that exhibited hyperphosphatemia and were undergoing MHD were enrolled for the present study. The patients were randomly divided into three groups: HD, n=23 patients; HDF, n=21 patients and HD+HP, n=21 patients. No significant differences regarding clinical and biological variables, including gender, age, dialysis vintage, URR, Kt/V, pre-treatment BUN levels, Scr, Pi or FGF-23 were observed in the three groups prior to treatment (Table I).

### Serum Pi levels

**Figure 1:**
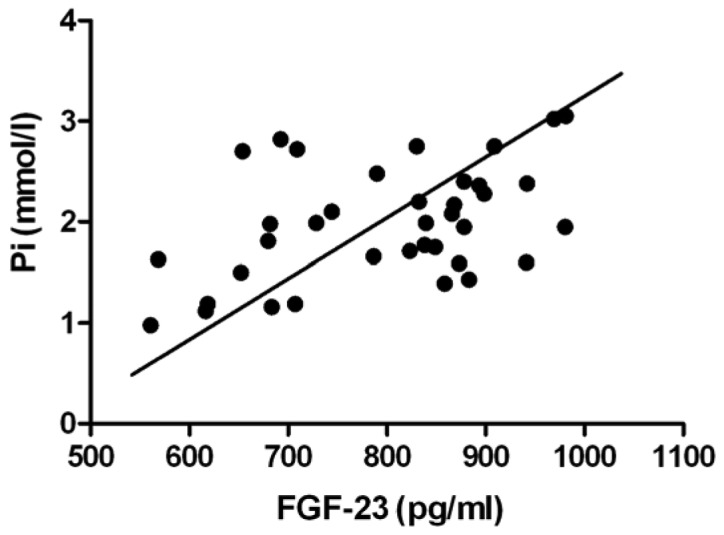
Correlation between serum levels of FGF-23 and Pi. The expression of serum FGF-23 was positively correlated with Pi (means ± standard deviation; Pearson product-moment correlation coefficient =0.45, P<0.01). FGF-23, fibroblast growth factor-23; Pi, phosphorus.

**Figure 2:**
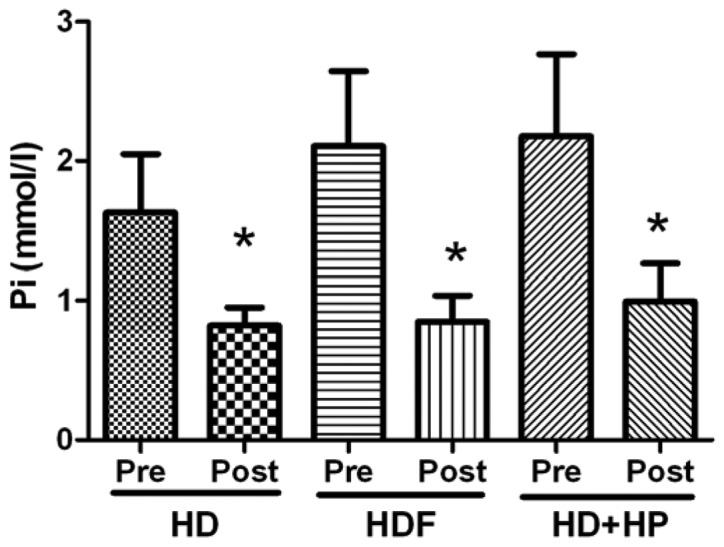
Expression of Pi in the patient serum. Serum Pi levels (means ± standard deviation) were significantly decreased post-treatment compared with pre-treatment in the three blood purification methods, respectively (^*^P<0.05). Pi, phosphorus; HD, hemodialysis; HDF, hemodiafiltration; HD+HP, hemodialysis and hemoperfusion.

In all of the specimens prior to treatment, the combined correlation analysis of serum FGF-23 levels indicated a positive correlation with serum Pi levels; the Pearson product-moment correlation coefficient was 0.45 and the difference was statistically significant (Fig. 1; P<0.01). The serum Pi levels of the HD group decreased from 1.63±0.42 mmol/l pre-hemodialysis to 0.82±0.13 mmol/l post-hemodialysis, the difference was identified to be statistically significant (Fig. 2; P<0.05). The HDF and HD+HP groups demonstrated significantly decreased serum Pi levels from 2.10±0.54 (pre-treatment) to 0.85±0.19 mmol/l (post-treatment; P<0.05) and from 2.18±0.59 (pre-treatment) to 0.99±0.27 mmol/l (post-treatment), respectively (Fig. 2; P<0.05).

### Pi reduction rate

**Figure 3:**
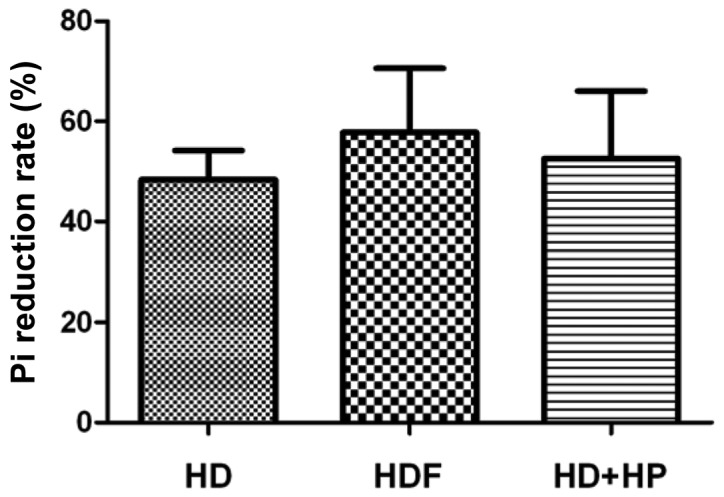
Pi reduction rate in the three groups. There was no statistical difference observed between the groups regarding the serum Pi reduction rate, P>0.05 (means ± standard deviation). Pi, phosphorus; HD, hemodialysis; HDF, hemodiafiltration; HD+HP, hemodialysis and hemoperfusion.

No statistically significant difference was identified between any of the groups regarding the Pi reduction rate in the three types of blood purification method (Fig. 3; P>0.05).

### Serum FGF-23 clearance

**Figure 4:**
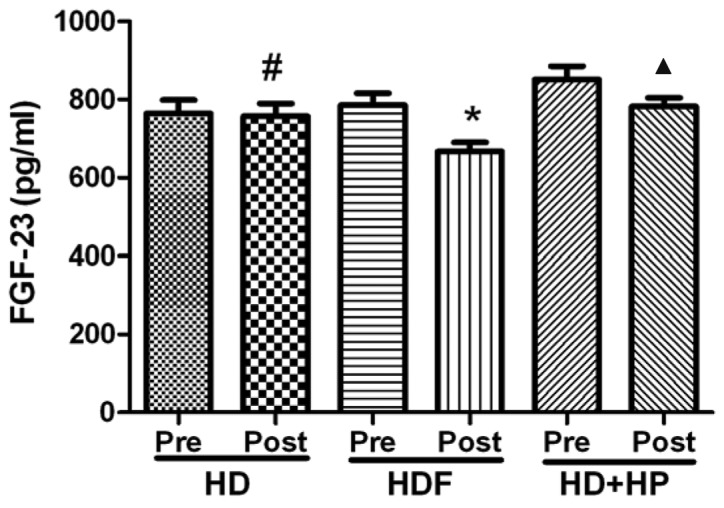
Serum FGF-23 clearance in patients pre- and post- treatment. The clearance of serum FGF-23 of the three blood purification method were decreased post-treatment compared with pre-treatment (means ± standard deviation). ^#^P>0.05, compared with pre-HD; ^*^P<0.05, compared with pre-HDF; ^▲^P<0.05, compared with pre-HD+HP. FGF-23, fibroblast growth factor-23; HD, hemodialysis; HDF, hemodiafiltration; HD+HP, hemodialysis and hemoperfusion.

The serum FGF-23 levels of the HD group decreased from 764.3±109.8 pg/ml pre-hemodialysis to 756.9±103.6 pg/ml post-hemodialysis, the difference was not identified to be significant (Fig. 4; P>0.05). The HDF and HD+HP groups demonstrated significant decreases in serum FGF-23 levels from 785.5±125.5 to 667.2±94.1 pg/ml (P<0.05) and from 850.9±108.6 to 782.2±71.9 pg/ml, respectively (Fig. 4; P<0.05).

### Comparison of blood purification methods

**Figure 5:**
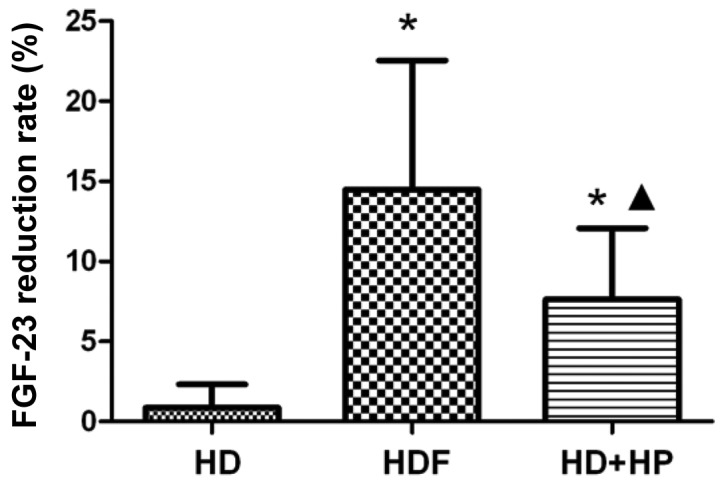
Comparison of the FGF-23 reduction rate between the three groups. There was a significant difference in the serum FGF-23 reduction rate between any two groups of the three blood purification method (means ± standard deviation). ^*^P<0.05, compared with HD; ^▲^P<0.05, compared with HDF. FGF-23, fibroblast growth factor 23; HD, hemodialysis; HDF, hemodiafiltration; HD+HP, hemodialysis and hemoperfusion.

A significant difference was observed in the FGF-23 reduction rate between any two of the three types of blood purification method (Fig. 5; P<0.05).

**Figure 6:**
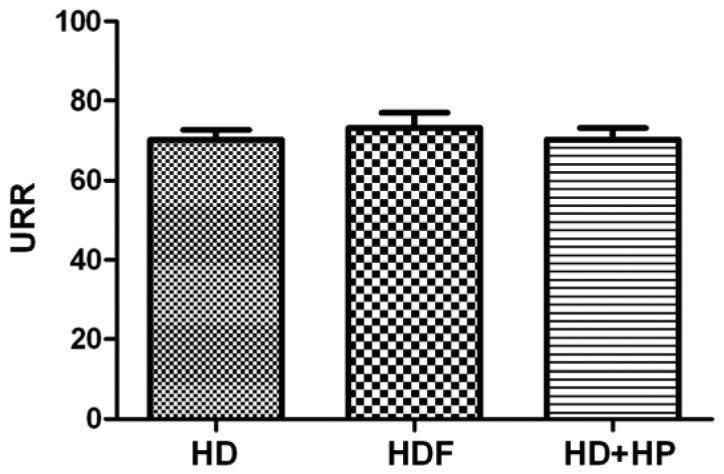
URR in the three groups. There was no significant differences observed between the groups in URR of three three blood purification methods, P>0.05 (means ± standard deviation). URR, urea reduction ratio; HD, hemodialysis; HDF, hemodiafiltration; HD+HP, hemodialysis and hemoperfusion.

A comparison of the URR of the three blood purification methods (HD, HDF and HD+HP) revealed values of: 70.27±2.55, 73.31±3.813 and 70.38±2.908, respectively; there were no statistically significant differences identified between any two of the three groups (Fig. 6; P>0.05).

**Figure 7:**
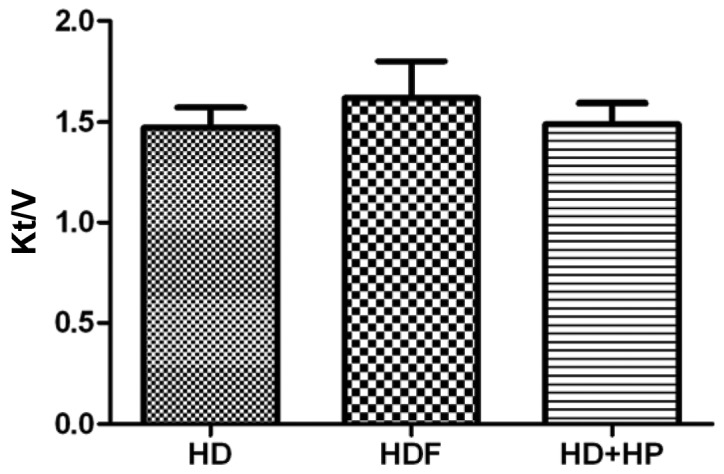
Urea clearance index (Kt/V) in the three groups. There was no statistical difference observed between the groups in the Kt/V of the three blood purification methods, P>0.05 (means ± standard deviation). HD, hemodialysis; HDF, hemodiafiltration; HD+HP, hemodialysis and hemoperfusion.

A comparison of the three blood purification methods (HD, HDF and HD+HP) regarding the Kt/V resulted in values of 1.473±0.099, 1.61±0.18 and 1.49±0.103, respectively, with no statistically significant differences observed between any two of the three groups (Fig. 7; P>0.05).

## Discussion

In the present study, three different blood purification methods were performed, HD, HDF and HD+HP, in patients that were exhibiting hyperphosphatemia and undergoing MHD.

The findings of the present study demonstrated a positive correlation between FGF-23 and Pi via a variable line correlation analysis of FGF-23 with pre-dialysis (Fig. 1). However, the underlying mechanism of this clinical manifestation remains unclear and may be associated with various factors. Perwad *et al* (17) conducted an *in vitro* study, which indicated that FGF-23 influenced 1a-hydroxylase expression via activation of the extracellular signal-regulated kinases-1/2 signaling pathway, which regulates the Pi levels. When kidney function declines, serum levels of FGF-23 increase, whereas the Pi values are not considered to be abnormal until the eGFR decreases to <30 ml/min/1.73 m^2^ (14). Thus, it has been hypothesized that there may be a negative feedback loop existing between FGF-23 levels and Pi disorders (9). Consistent with these findings, an injection of the FGF23 C-terminal tail peptide in healthy rats was shown to inhibit renal phosphate excretion and induce hyperphosphatemia (18). The results of the present study were consistent with previous studies (9,14,17,18) and demonstrated that high levels of FGF-23 were closely associated with hyperphosphatemia in MHD patients. Therefore, cleaning the FGF-23 of the MHD patients, and then reducing hyperphosphatemia due to elevated serum FGF-23 level, and ultimately resulting in improvement of the long-term prognosis of patients, are the important issues of blood purification.

In the present study, the three blood purification methods were effective in the clearance of Pi (Fig. 2; P<0.05); however, there were no statistically significant differences identified between the groups regarding the Pi reduction rate (Fig. 3; P>0.05), which demonstrated that the efficacy of the three Pi clearance methods was similar. However, the use of blood purification for Pi removal is not considered to be sufficient as 14% of Pi is present in the intracellular fluid and l% is present in the extracellular fluid; thus, Pi release from the cells into the blood occurs more slowly compared with Pi removal during dialysis. Therefore, the treatment of hyperphosphatemia clinically controls Pi levels, however, is also required to control the serum Ca, PTH and FGF-23 levels, as dietary Pi restriction and Pi removal via blood purification alone are insufficient.

In the present study, the efficacy of clearing serum FGF-23 using three blood purification methods in MHD patients was compared. It was identified that HD was not able to clear FGF-23 but HDF and HD+HP were able to clear FGF-23 effectively, however, HDF exhibited the optimum efficacy (Figs. 4 and 5).

FGF-23 is a medium-sized molecule (19); HD was able to clear the small molecule toxins, such as urea, nitrogen and creatinine via dispersion, however, the molecular weight cut-offs of the HD membranes were <5 kDa, therefore removal of important intact FGF-23 (molecular weight, 32,000) was not possible. This was consistent with a previous study by Urena Torres *et al* (20).

HDF is a type of blood purification technique, which combines diffusion and convection. Convection is the primary method of clearing macromolecules and the convective clearance rate is predominantly dependent on the transmembrane pressure and the ultrafiltration coefficient of the dialyzer. The maximum transmembrane pressure of the dialyzer that was used in the present study was 600 mmHg with an ultrafiltration coefficient of 50 ml/h mmHg. Taken together, these factors validate the predominant role that convection has in the removal of medium molecular weight molecules, such as FGF-23. However, in the present study, the clearing effect of FGF-23 in the HDF group was lower than the values reported by Patrier *et al* (21). This may be due to the HDF treatment effect being dose-dependent and the filter ultrafiltration coefficient, membrane area, blood flow and displacement liquid, which were used in the present study, being lower than those that were reported in previous studies.

HP involves extraction of the patient’s blood and the harmful metabolites are subsequently removed through a neutral macroporous resin apparatus, *in vitro*. The HA130-type blood perfusion resin that was used in the present study is a novel neutral synthetic resin with an average pore diameter of 13–15 nm and a specific surface area of ≤1000–1500 m^2^/g. Due to physical adsorption and the interaction of the hydrophobic group, the resin strongly and non-specifically adsorbs medium-sized molecules, such as FGF-23. The adsorbent is not able to effectively remove water, urea, nitrogen or other small molecules, therefore, the adsorption treatment in MHD patients often uses a combination of HD+HP blood purification methods to completely remove the metabolic waste products.

In the present study, the clearance of FGF-23 in the HD+HP group was observed to be less effective than that of the HDF group. A possible mechanism may be related to the furin proteolytic enzyme cleavage of 179 arginine/180 serine within FGF-23, which may have been separated into N-terminal (18 kDa) and C-terminal (12 kDa) fragments. The distribution of varying molecular weights and different forms of FGF-23 in the blood circulation is unknown. Therefore, convection by HDF is considered to be more effective than adsorption by HP for the clearance of FGF-23 (18).

Comparisons between the three blood purification methods regarding URR and the Kt/V indicated no statistically significant differences. Therefore, it was hypothesized that the difference between the three blood purification methods regarding serum FGF-23 clearance was not due to the adequacy of dialysis (Figs. 6 and 7).

In conclusion, the expression of serum FGF-23 was positively correlated with the levels of Pi in MHD patients exhibiting hyperphosphatemia. In addition, FGF-23 was identified to be an important regulator of the clearance of Pi from the blood. However, a limitation of the present study was that the long-term clinical significance of the different blood purification methods was not observed, therefore, future studies are required for further investigation.

## Figures and Tables

**Table I:** Clinical and biological variables of the three groups prior to treatment.

Variable	HD (n=23)	HDF (n=21)	HD+HP (n=21)	Statistical significance
Males, n (%)	12 (52.2)	13 (61.9)	12 (57.1)	#P
Age (years)	42.5±12.7	42.2±17.5	48.1±15.8	*P
Dialysis vintage (months)	41.2±22.6	48.1±27.1	54.4±25.3	*P
Urea reduction ratio (%)	70.27±2.55	73.31±3.813	70.38±2.908	*P
Kt/V	1.473±0.099	1.61±0.18	1.49±0.103	*P
Pre-treatment BUN (mmol/l)	25.2±6.2	27.3±4.8	25.1±4.1	*P
Pre-treatment SCr (μmol/l)	1024±199.6	1103±230.7	1030±180.6	*P
Pre-treatment Pi (mmol/l)	1.63±0.42	2.10±0.54	2.18±0.59	*P
Pre-treatment FGF-23 (pg/ml)	764.3±109.8	785.5±125.5	850.9±108.6	*P

# P>0.05, χ^2^-test;

* P>0.05, one-way analysis of variance.

Data are presented as means ± standard deviation. There was no statistical difference identified between gender, age, dialysis vintage, urea reduction ratio, Kt/V, pre-treatment BUN levels, Scr, Pi, and FGF-23, between any two of the three groups. HD, hemodialysis group; HDF, hemodiafiltration group; HD+HP, hemodialysis+hemoperfusion group; BUN, blood urea nitrogen; SCr, serum creatinine; Pi, phosphorous; FGF-23, fibroblast growth factor-23.

## References

[1] National Kidney Foundation (2002). K/DOQI clinical practice guidelines for chronic kidney disease: evaluation, classification, and stratification. Am J Kidney Dis.

[2] Vassalotti JA, Stevens LA, Levey AS (2007). Testing for chronic kidney disease: a position statement from the National Kidney Foundation. Am J Kidney Dis.

[3] Stevens LA, Levey AS (2009). Current status and future perspectives for CKD testing. Am J Kidney Dis.

[4] Levey AS, Coresh J (2012). Chronic kidney disease. Lancet.

[5] Hsu CY, Ordoñez JD, Chertow GM, Fan D, McCulloch CE, Go AS (2008). The risk of acute renal failure in patients with chronic kidney disease. Kidney Int.

[6] James MT, Hemmelgarn BR, Wiebe N, Alberta Kidney Disease Network (2010). Glomerular filtration rate, proteinuria, and the incidence and consequences of acute kidney injury: a cohort study. Lancet.

[7] James MT, Quan H, Tonelli M, Alberta Kidney Disease Network (2009). CKD and risk of hospitalization and death with pneumonia. Am J Kidney Dis.

[8] Hailpern SM, Melamed ML, Cohen HW, Hostetter TH (2007). Moderate chronic kidney disease and cognitive function in adults 20 to 59 years of age: Third National Health and Nutrition Examination Survey (NHANES III). J Am Soc Nephrol.

[9] Miyamoto K, Ito M, Tatsumi S, Kuwahata M, Segawa H (2007). New aspect of renal phosphate reabsorption: the type IIc sodium-dependent phosphate transporter. Am J Nephrol.

[10] Hu P, Xuan Q, Hu B, Lu L, Wang J, Qin YH (2012). Fibroblast growth factor-23 helps explain the biphasic cardiovascular effects of vitamin D in chronic kidney disease. Int J Biol Sci.

[11] Slatopolsky E, Moe S (2011). 50 years of research and discovery in chronic kidney disease and mineral & bone disorder: the central role of phosphate. Kidney Int Suppl.

[12] Shigematsu T, Nakashima Y, Ohya M (2012). The management of hyperphosphatemia by lanthanum carbonate in chronic kidney disease patients. Int J Nephrol Renovasc Dis.

[13] Gutierrez O, Isakova T, Rhee E (2005). Fibroblast growth factor-23 mitigates hyperphosphatemia but accentuates calcitriol deficiency in chronic kidney disease. J Am Soc Nephrol.

[14] Bia M, Adey DB, Bloom RD, Chan L, Kulkarni S, Tomlanovich S (2010). KDOQI US commentary on the 2009 KDIGO clinical practice guideline for the care of kidney transplant recipients. Am J Kidney Dis.

[15] Noordzij M, Korevaar JC, Boeschoten EW, Netherlands Cooperative Study on the Adequacy of Dialysis (NECOSAD) Study Group (2005). The Kidney Disease Outcomes Quality Initiative (K/DOQI) Guideline for Bone Metabolism and Disease in CKD: association with mortality in dialysis patients. Am J Kidney Dis.

[16] Daugirdas JT (1993). Second generation logarithmic estimates of single-pool variable volume Kt/V: an analysis of error. J Am Soc Nephrol.

[17] Perwad F, Zhang MY, Tenenhouse HS, Portale AA (2007). Fibroblast growth factor 23 impairs phosphorus and vitamin D metabolism in vivo and suppresses 25-hydroxyvitamin D-1alpha-hydroxylase expression in vitro. Am J Physiol Renal Physiol.

[18] Goetz R, Nakada Y, Hu MC (2010). Isolated C-terminal tail of FGF23 alleviates hypophosphatemia by inhibiting FGF23-FGFR-Klotho complex formation. Proc Natl Acad Sci USA.

[19] Chen HC, Lim LM, Chang JM, Misra M (2013). Save life and improve quality: Report from the 5th Congress of International Society for Hemodialysis. Hemodial Int.

[20] Urena Torres P, Friedlander G, de Vernejoul MC, Silve C, Prié D (2008). Bone mass does not correlate with the serum fibroblast growth factor 23 in hemodialysis patients. Kidney Int.

[21] Patrier L, Dupuy AM, Granger Vallée A (2013). FGF-23 removal is improved by on-line high-efficiency hemodiafiltration compared to conventional high flux hemodialysis. J Nephrol.

